# ASK1 restores the antiviral activity of APOBEC3G by disrupting HIV-1 Vif-mediated counteraction

**DOI:** 10.1038/ncomms7945

**Published:** 2015-04-22

**Authors:** Kei Miyakawa, Satoko Matsunaga, Kazuhiko Kanou, Atsushi Matsuzawa, Ryo Morishita, Ayumi Kudoh, Keisuke Shindo, Masaru Yokoyama, Hironori Sato, Hirokazu Kimura, Tomohiko Tamura, Naoki Yamamoto, Hidenori Ichijo, Akifumi Takaori-Kondo, Akihide Ryo

**Affiliations:** 1 Department of Microbiology, Yokohama City University School of Medicine, Kanagawa 236-0004, Japan; 2 Infectious Disease Surveillance Center, National Institute of Infectious Diseases, Tokyo 162-8640, Japan; 3 Laboratory of Cell Signaling, Graduate School of Pharmaceutical Sciences, The University of Tokyo, Tokyo 113-0033, Japan; 4 CellFree Sciences Co. Ltd., Ehime University Venture, Ehime 790-8577, Japan; 5 Department of Hematology and Oncology, Graduate School of Medicine, Kyoto University, Kyoto 606-8507, Japan; 6 Pathogen Genomics Center, National Institute of Infectious Diseases, Tokyo 208-0011, Japan; 7 Department of Immunology, Yokohama City University School of Medicine, Kanagawa 236-0004, Japan; 8 Department of Microbiology, National University of Singapore, Singapore 117597, Singapore

## Abstract

APOBEC3G (A3G) is an innate antiviral restriction factor that strongly inhibits the replication of human immunodeficiency virus type 1 (HIV-1). An HIV-1 accessory protein, Vif, hijacks the host ubiquitin–proteasome system to execute A3G degradation. Identification of the host pathways that obstruct the action of Vif could provide a new strategy for blocking viral replication. We demonstrate here that the host protein ASK1 (apoptosis signal-regulating kinase 1) interferes with the counteraction by Vif and revitalizes A3G-mediated viral restriction. ASK1 binds the BC-box of Vif, thereby disrupting the assembly of the Vif–ubiquitin ligase complex. Consequently, ASK1 stabilizes A3G and promotes its incorporation into viral particles, ultimately reducing viral infectivity. Furthermore, treatment with the antiretroviral drug AZT (zidovudine) induces ASK1 expression and restores the antiviral activity of A3G in HIV-1-infected cells. This study thus demonstrates a distinct function of ASK1 in restoring the host antiviral system that can be enhanced by AZT treatment.

## Abstract (web summary)

The human protein APOBEC3G (A3G) inhibits HIV-1 replication, but the viral protein Vif counteracts by inducing A3G degradation. Here Miyakawa *et al*. show that the antiretroviral drug AZT restores A3G function *in vitro* by stimulating expression of a host protein, ASK1, which interferes with the action of Vif.

## Graphical abstract

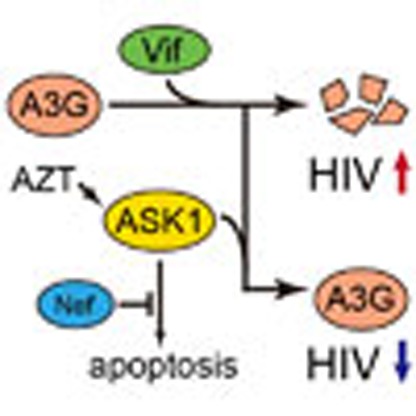

The innate immune system is an evolutionarily conserved network that acts as a first-line defense against invading microbial pathogens and other potential threats to host cells^1^. In addition to the nonspecific or broadly specific counteraction exerted by the physiological component of innate immunity, a more specific response is exerted by intracellular restriction factors, which belong to a group of interferon-stimulated genes^2^,^3^. When interferons induce their transcription, restriction factors limit the replication of invading viruses. One such factor is an editing enzyme for nucleic acids, APOBEC3G (apolipoprotein B mRNA-editing enzyme catalytic polypeptide-like 3G, hereafter referred to as A3G). This protein severely restricts the replication of numerous viruses, including human immunodeficiency virus type 1 (HIV-1)^4^ and hepatitis B virus^5^, by extensively deaminating cytosine residues in the viral genome during reverse transcription. This process introduces unnatural (cytosine-to-uracil) mutations in the minus-strand viral DNA, leading to either the destabilization of reverse transcripts or the production of defective viral proteins^6^,^7^,^8^. In addition, A3G appears to inhibit the elongation of reverse transcripts by deaminase-independent mechanisms^9^,^10^.

Although A3G is a potent antiviral molecule, HIV-1 has developed a specific accessory protein, Vif, which can counteract the antiviral activity of A3G. In infected cells, Vif forms an ubiquitin ligase complex with Cullin5 (CUL5), Elongin B/C (ELOB/C) and CBFβ that ubiquitinates and degrades A3G^11^,^12^,^13^. In HIV-1 isolates lacking the Vif gene, A3G is efficiently incorporated into virions by interacting with viral nucleocapsid protein and viral RNA, severely limiting viral replication in the target cells^14^,^15^. In addition, many studies using CD4^+^ lymphocytes or humanized mice suggest that A3G activity is crucial for inhibiting viral replication and pathogenesis^4^,^16^,^17^.

Thus, the strategies to promote A3G stability and its incorporation into virions could be a new avenue for the development of new antiviral drugs. In this regard, the disruption of any of the steps required for the effect of Vif on A3G would be expected to restore cellular A3G levels and virus restriction. This concept has been validated in several reports that used a fluorescence-based screen to identify a small compound that specifically inhibits the Vif–A3G interaction^18^,^19^,^20^. However, it is still unclear if Vif is regulated by external or internal cellular signalling and which cellular components are involved. Thus, the identification of host regulators of Vif may provide an alternative therapeutic strategy for HIV-1 infection that preserves the antiviral activity of the host defense system.

Here we demonstrate that apoptosis signal-regulating kinase 1 (ASK1) binds ‘hot spots' within Vif to block its interaction with components forming the ubiquitin ligase complex, resulting in the stabilization of A3G and reactivation of A3G-mediated host defense activity. We have therefore identified a novel host factor governing the Vif–A3G interaction that directs the restoration of the innate antiviral response.

## Results

### ASK1 binds and counteracts Vif

**Figure 1:**
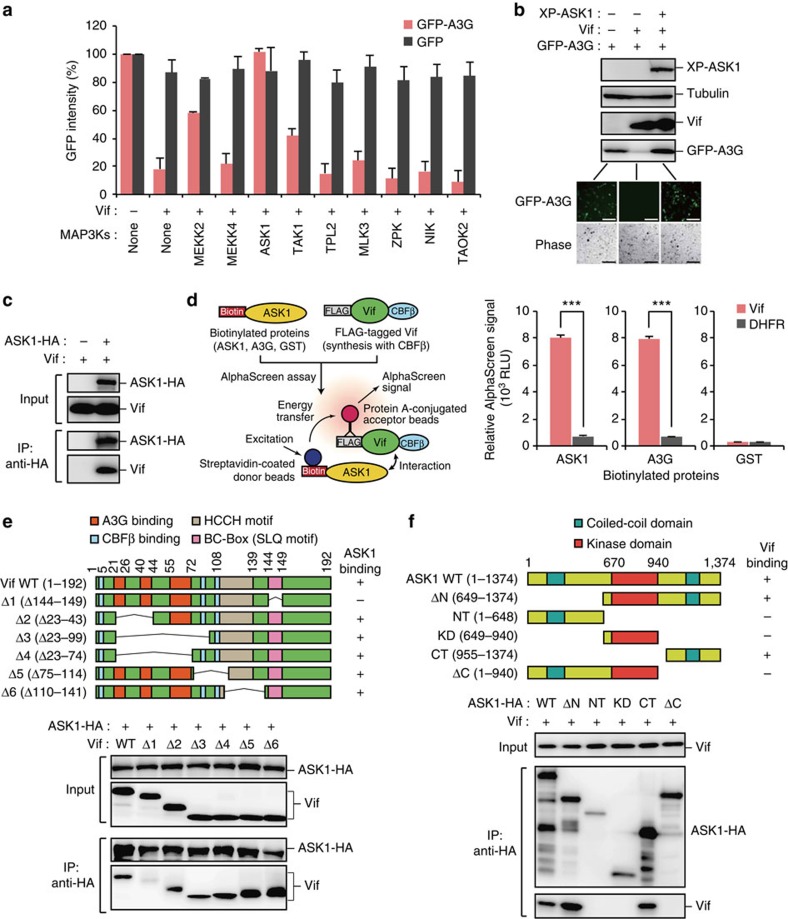
ASK1 binds and counteracts Vif. (**a**,**b**) ASK1 inhibits Vif-mediated A3G degradation. HEK293 cells were cotransfected with plasmids encoding Vif (250 ng), GFP-A3G (50 ng) and the indicated HA-tagged MAP3Ks or Xpress (XP)-tagged ASK1 (500 ng). After 48 h, GFP intensities (*n*=3, mean±s.d.), cell images and protein expression levels were analysed by flow cytometry (**a**), fluorescence microscopy and western blotting (**b**), respectively. Scale bar, 100 μm. (**c**) ASK1 interacts with Vif in cells. Cells were cotransfected with plasmids encoding ASK1-HA and Vif. Cell lysates were immunoprecipitated with an anti-HA antibody and the bound proteins were then analysed by western blotting. (**d**) *In vitro* interaction of ASK1 with Vif. Schematic representation of the amplified luminescent proximity homogeneous (AlphaScreen) assay used to detect the direct protein–protein interaction (left). In this study, FLAG-tagged Vif proteins were co-expressed with CBFβ to stabilize the conformation of the Vif protein^28^,^63^. AlphaScreen analysis (*n*=3, mean±s.d.) using recombinant ASK1, A3G and Vif proteins is shown (right). GST and DHFR (dihydrofolate reductase) were used as negative controls. ***P<0.001, two-tailed unpaired *t*-test. (**e**) HEK293 cells were cotransfected with plasmids encoding ASK1-HA and Vif or one of its truncated mutants (top). Cell lysates were immunoprecipitated with anti-HA antibody and bound Vif proteins were detected with western blot analysis (bottom). (**f**) HEK293 cells were cotransfected with plasmids encoding Vif and ASK1-HA or one of its truncated mutants (top). Cell lysates were immunoprecipitated with anti-HA antibody and bound Vif proteins were detected by western blot analysis (bottom). Full images for all western blots analysis are shown in Supplementary Fig. 5.

The mitogen-activated protein (MAP) kinase signalling pathway can transduce extracellular signals into regulatory events that impact the responses of cells to such stimuli^21^. The kinase cascade eventually modulates the cellular context, leading to the regulation of transcription factors affecting gene expression. MAP3Ks are regarded as effectors of the recognition of a variety of stimuli and activators of intracellular signal transduction pathways^22^,^23^,^24^. We thus initially determined whether MAP3K family members could affect the Vif-mediated counteraction of A3G. HEK293 cells were cotransfected with plasmids encoding Vif, green fluorescent protein (GFP)-A3G, and the indicated MAP3Ks, and then GFP intensities were assessed with flow cytometry and immunoblot analysis. Notably, the expression of MAP3K5, also named ASK1, maintained A3G expression, even in the presence of Vif (Fig. 1a,b). Immunoprecipitation analysis showed that ASK1 interacted with Vif (Fig. 1c). This interaction was also confirmed by an *in vitro* protein–protein interaction assay with the amplified luminescent proximity homogenous assay AlphaScreen^25^ (Fig. 1d). Moreover, the activation or suppression of ERK-, p38- and JNK-mediated MAPK pathways did not alter the ability of ASK1 to bind and counteract Vif (Supplementary Fig. 1a–c). These results suggest that ASK1 directly binds Vif and suppresses the action of Vif on A3G. We next sought to identify the binding regions within Vif for its interaction with ASK1. As well as an A3G-binding domain in the amino (N)-terminal half, Vif contains an HCCH motif and a BC-box (SLQ motif) in the carboxyl (C)-terminal half that are essential for interaction with CUL5 and ELOB/C, respectively^26^,^27^. The N-terminal and central domains of Vif are involved in binding CBFβ^12^,^27^,^28^,^29^. Immunoprecipitation experiments with truncated mutants of Vif showed that ASK1 associated with all Vif mutants except the VifΔ1 mutant lacking the BC-box (Fig. 1e). Notably, ASK1 failed to interact with another BC-box-containing protein VHL (von Hippel–Lindau)^30^ and did not affect the function of VHL in downregulating hypoxia-inducible factor 1-α (Supplementary Fig. 2a,b). This implied a specific interaction of ASK1 with Vif. We next mapped the Vif-binding site within ASK1. ASK1 contains a kinase domain in the central region and two coiled-coil domains in the N- and C-terminal regions^31^. Immunoprecipitation analysis with truncated mutants of ASK1 clearly showed that Vif binds the C-terminal (CT: 955–1,374 amino acids) domain of ASK1 (Fig. 1f). Our results thus suggested that the ASK1 CT may interact with the BC-box of HIV-1 Vif.

### ASK1 inhibits the formation of Vif–E3 ubiquitin ligase complex

**Figure 2:**
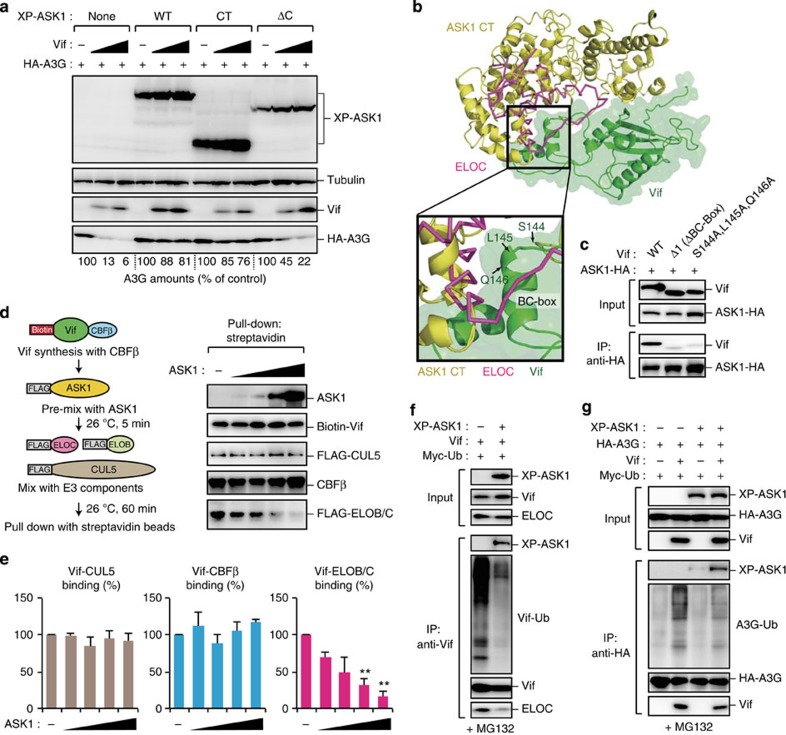
ASK1 inhibits the formation of Vif–E3 ubiquitin ligase complex. (**a**) The C-terminal domain (CT) of ASK1 is sufficient to impair Vif-mediated A3G degradation. HEK293 cells were cotransfected with plasmids encoding XP-ASK1 (500 ng), HA-A3G (10 ng) and Vif (50 or 100 ng). Protein expression was then detected using western blot analysis. The numerical values below the blot indicate the amounts of HA-A3G determined with densitometry. (**b**) The structural model of ASK1 CT (yellow) was generated and subjected to docking simulation with Vif (green, PDB: 4N9F). ELOC (purple) was overlaid onto the Vif–ASK1 model. The square shows the predicted inhibition by ASK1 of ELOC binding to the BC-Box of Vif. (**c**) The BC-box motif of Vif is important for the binding of ASK1. HEK293 cells were cotransfected with plasmids encoding ASK1-HA and Vif or the indicated mutants. Cell lysates were immunoprecipitated with anti-HA antibody and bound Vif proteins were detected by western blot analysis. (**d**,**e**) ASK1 inhibits the formation of the ubiquitin ligase complex by blocking Vif interaction with ELOB/C. (**d**) Recombinant biotinylated Vif was co-synthesized with CBFβ and then pre-mixed with various amounts of ASK1 for 5 min before the addition of equivalent amounts of FLAG-tagged CUL5, ELOB and ELOC. After 1 h, the mixture was processed for pull down with streptavidin-coated magnetic beads as shown on the left. Bound proteins were detected by western blot (right panels). The bar charts shown in **e** indicate the amounts of Vif-associated proteins in the presence of ASK1, determined by densitometric analysis of the western blots (*n*=3, mean±s.d.). **P<0.01, two-tailed unpaired *t*-test. (**f**) HEK293 cells were cotransfected with plasmids encoding XP-ASK1, Vif and Myc-Ubiquitin (Ub). Cells were then treated with MG132 for 18 h before being harvested. Cell lysates were immunoprecipitated with anti-Vif antibody and the ligase activity of Vif was detected by western blot analysis using an anti-Myc antibody. (**g**) HEK293 cells were cotransfected with plasmids encoding XP-ASK1, HA-A3G, Vif and Myc-Ub. Cells were then treated with MG132 for 18 h before being harvested. Cell lysates were immunoprecipitated with anti-HA antibody and the Vif-induced ubiquitination of A3G was detected by western blot analysis using anti-Myc antibody. Full images for all western blots analysis are shown in Supplementary Fig. 5.

We next assessed the functional and structural aspects of the Vif–ASK1 interaction. Consistent with the results of our binding analysis (Fig. 1f), ASK1 CT was sufficient to inhibit Vif-mediated A3G degradation, whereas an ASK1 ΔC mutant devoid of the Vif-binding domain had lost this ability (Fig. 2a). To predict the functional impact of the binding of ASK1 to Vif, we constructed ASK1 CT structural models and docked them with the recently solved Vif structure (PDB: 4N9F). Interestingly, our computational docking simulation data proposed a model whereby ASK1 CT interacts with the BC-box of Vif (Fig. 2b), consistent with our immunoprecipitation results (Fig. 1e,f). This structural model also predicted that ASK1 CT–Vif binding might partially interfere with the association of Vif with ELOC (Fig. 2b). Indeed, Vif BC-box mutants (S144A, L145A, and Q146A) that demonstrate impaired binding of ELOC^32^, but not Vif mutants of other potential ASK1-binding sites (R93, Y94, I124 and L125), failed to interact with ASK1 (Fig. 2c, Supplementary Fig. 3). To further test this hypothesis, we performed *in vitro* pull-down analysis with recombinant versions of the proteins forming the Vif-mediated E3 complex (CUL5, ELOB/C and CBFβ) in the presence or absence of ASK1. FLAG-tagged CUL5, ELOB/C and CBFβ proteins were mixed with biotin-labelled Vif and various amounts of ASK1, and then Vif-interacting components were pulled-down using streptavidin-coated beads (Fig. 2d). Subsequent immunoblotting analysis demonstrated that ASK1 inhibited the interaction of Vif with ELOB/C in a dose-dependent manner, whereas no significant change was observed in the binding of Vif with either CUL5 or CBFβ (Fig. 2d,e). Consistent with these *in vitro* interaction data, our cell-based analysis further revealed that ASK1 reduced the E3 ligase activity of Vif (including the autoubiquitination of Vif), which was accompanied by a reduction in the Vif–ELOC interaction (Fig. 2f). Moreover, ASK1 markedly reduced Vif ubiquitination of A3G (Fig. 2g). Although Mehle *et al*.^33^ have reported previously that phosphorylation of the Vif BC-box negatively regulates Vif–ELOB/C complex assembly, we could not detect any Vif phosphorylation by ASK1 (Supplementary Fig. 4). Our data suggest that ASK1 interacts with the BC-box motif of Vif and inhibits the formation of the E3 complex by interfering with the interaction between Vif and ELOB/C.

### Nef does not affect ASK1-mediated Vif inactivation

**Figure 3:**
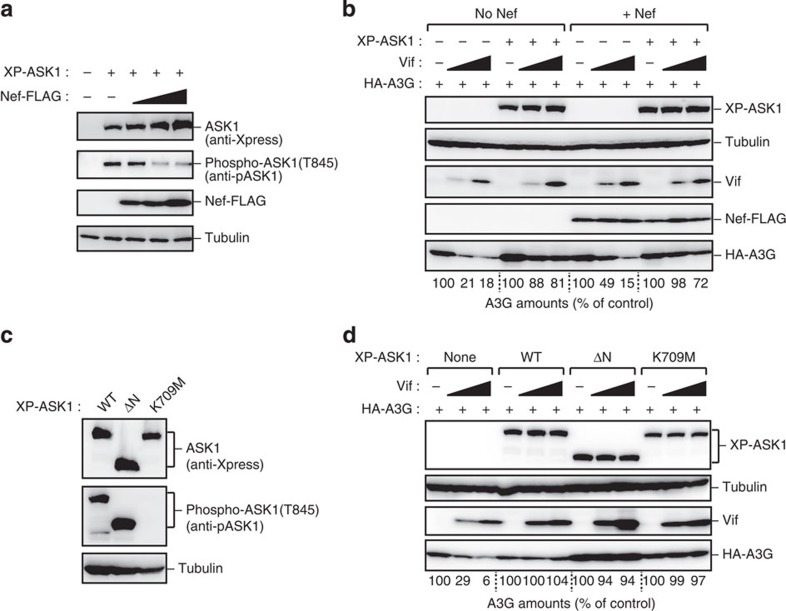
Nef does not affect ASK1-mediated Vif inactivation. (**a**) Nef inhibits the autophosphorylation of ASK1. HEK293 cells were cotransfected with plasmids encoding XP-ASK1 (100 ng) and Nef-FLAG (100 or 200 ng). Cell lysates were subjected to western blot analysis using the indicated antibodies. (**b**) Nef does not alter the effect of ASK1 on Vif-mediated A3G degradation. HEK293 cells were cotransfected with plasmids encoding XP-ASK1 (500 ng), HA-A3G (10 ng), Vif (50 or 100 ng) and Nef-FLAG (1 μg). Protein expression was detected by western blot. The numerical values below the blot indicate the amounts of HA-A3G determined with densitometry. (**c**) HEK293 cells were transfected with the indicated ASK1 mutants (ΔN, constitutively active; K709M, kinase-dead). Cell lysates were subjected to western blotting against the indicated antibodies. (**d**) HEK293 cells were cotransfected with plasmids encoding HA-A3G (10 ng), Vif (50 or 100 ng) and with wild-type ASK1 or one of its kinase mutants (500 ng). Protein expression was detected by western blot. The numerical values below the blot indicate the amounts of HA-A3G as determined by densitometry. Full images for all western blots analysis are shown in Supplementary Fig. 5.

Previous studies have demonstrated that ASK1 potently associates with Nef, another HIV accessory protein. Nef reduces the kinase activity of ASK1 to prevent tumour necrosis factor-α- and FAS-dependent apoptosis^34^. We thus investigated whether Nef affects ASK1-mediated Vif inactivation. Immunoblotting analysis revealed that Nef overexpression inhibited the autophosphorylation of ASK1 (phosphorylated Thr845 of ASK1), a hallmark of its kinase activity (Fig. 3a). Notably, irrespective of Nef expression, our data also showed that ASK1 effectively inhibited Vif-mediated A3G degradation (Fig. 3b), suggesting that the kinase activity of ASK1 is dispensable for its ability to inhibit Vif. Moreover, a kinase-negative (K709M) mutant of ASK1 also inhibited Vif-mediated A3G degradation, although a constitutively kinase-active ASK1 (ΔN) mutant exhibited a slightly higher ability to inhibit Vif via an unknown mechanism (Fig. 3c,d). These results suggest that ASK1 kinase activity is dispensable for, but has an additive effect in, inhibiting the function of Vif. This is indicative of the involvement of multiple mechanisms in the ASK1-mediated inhibition of Vif.

### ASK1 restricts HIV-1 replication via A3G reactivation

**Figure 4:**
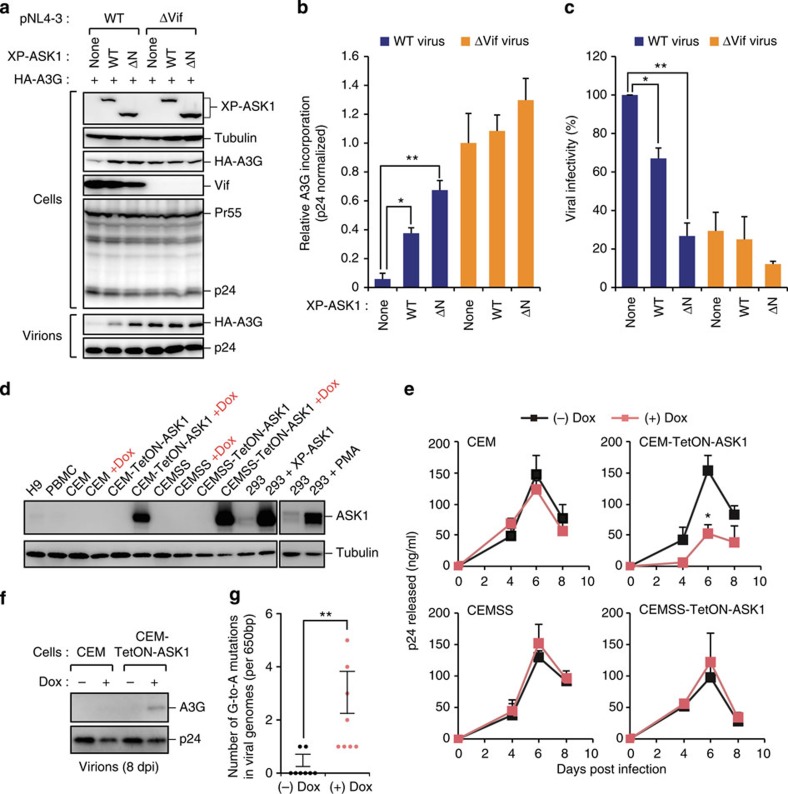
ASK1 restricts HIV-1 replication via A3G reactivation. (**a**–**c**) ASK1 expression in virus-producing cells promotes A3G incorporation into virions and reduces infectivity. HEK293 cells were cotransfected with an HIV-1 molecular clone carrying a GFP reporter gene (pNL4-3ΔEnv-GFP) or its Vif-deficient mutant (pNL4-3ΔEnvΔVif-GFP) together with a plasmid encoding VSV-G, XP-ASK1 and HA-A3G. (**a**,**b**) Forty-eight hours after transfection, cell lysates and supernatants were harvested and analysed by western blotting against the indicated antibodies. The bar chart in **b** indicates the amounts of HA-A3G normalized by p24 levels in virions, as determined by densitometric analysis of western blots (*n*=3, mean±s.d.). (**c**) The CD4^+^ T-cell line (M8166) was infected with harvested and normalized virus for 2 days and infected (GFP-positive) cells were then measured by flow cytometry (*n*=3, mean±s.d.). *P<0.05; **P<0.01, two-tailed unpaired *t*-test. (**d**) Expression levels of ASK1 in DOX-treated or untreated CEM-TetON-ASK1 and CEMSS-TetON-ASK1 cells. 293 cells transfected with ASK1 or treated with PMA are shown as positive controls. (**e**–**g**) CEM-TetON-ASK1 and CEMSS-TetON-ASK1 cells and their parent cell lines (CEM and CEMSS) were infected with HIV-1. (**e**) Culture supernatants were harvested at the indicated time-points and subjected to p24 ELISA (*n*=3, mean±s.d.). *P<0.05, two-tailed unpaired *t*-test. (**f**) The incorporation of A3G into virions from indicated cells (at 8 d.p.i.) was detected by western blot. (**g**) The infected cells (CEM-TetON-ASK1) were harvested at 8 d.p.i. and subjected to G-to-A hypermutation analysis (*n*=8, mean±s.d.). **P<0.01, two-tailed unpaired *t*-test. Full images for all western blots analysis are shown in Supplementary Fig. 6. d.p.i., days post infection; ELISA, enzyme-linked immunosorbent assay.

To test whether ASK1 regulates viral infectivity by interfering with Vif function and stabilizing A3G, we performed a single-cycle viral infection assay using HIV-1_NL4-3_ and its Vif-deficient mutant virus collected from ASK1-expressing cell supernatants. Immunoblotting analysis of cell lysates and viral supernatants revealed that the expression of either ASK1 or its ΔN mutant suppressed the Vif-mediated degradation of A3G in cells, increasing the amount of A3G in virions to that seen with a Vif-deficient virus (Fig. 4a,b). Consistent with this result, the infectivity of viruses harvested from either ASK1- or ASK1 ΔN-overexpressing cells was much lower than that of control cells (Fig. 4c). Since the endogenous ASK1 levels in T cells were nearly undetectable in the normal state (Fig. 4d), we generated stable cell lines, CEM (A3G-positive) and CEMSS (A3G-negative), harbouring a tetracycline-inducible ASK1 gene, referred to hereafter as CEM-TetON-ASK1 and CEMSS-TetON-ASK1, respectively. Treatment with a tetracyclic antibiotic, doxycycline (Dox), induced the expression of ASK1 in both cell lines at the physiological levels seen in PMA-treated 293 cells^35^ (Fig. 4d). Notably, Dox-induced ASK1 inhibited HIV-1 replication in CEM-TetON-ASK1 but not in CEMSS-TetON-ASK1 cells (Fig. 4e). Moreover, immunoblotting analysis demonstrated that the A3G level in virions was increased only in Dox-treated CEM-TetON-ASK1 cells (Fig. 4f). In addition, the number of G-to-A hypermutations in the viral genomes was markedly increased in these cells (Fig. 4g). Taken together, our data suggest that ASK1 restricts the replication of HIV-1 by promoting A3G incorporation into virions in human CD4^+^ T cells.

### AZT induces ASK1 and promotes the antiviral activity of A3G

**Figure 5:**
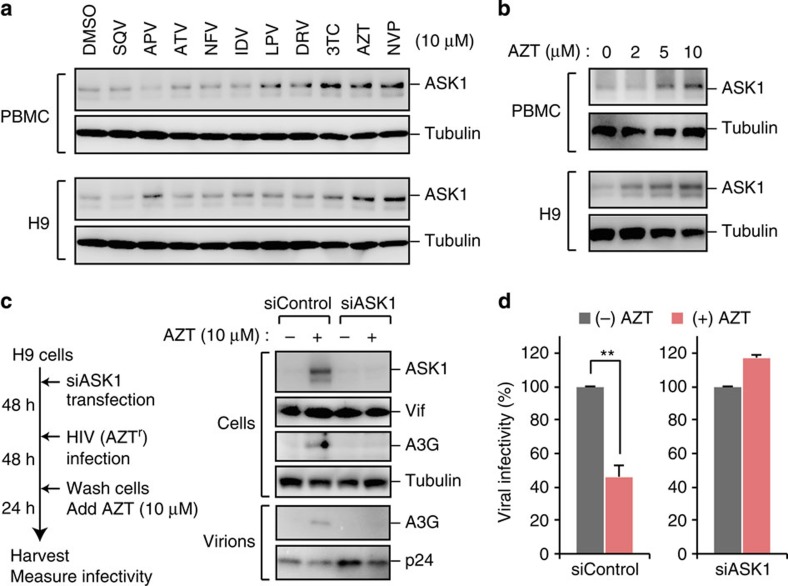
AZT induces ASK1 and promotes the antiviral activity of A3G. (**a**,**b**) PBMC or H9 cells were treated with the indicated agents (0–10 μM) for 24 h. The expression of ASK1 was confirmed by western blotting. APV, amprenavir; ATV, atazanavir; AZT, zidovudine; DRV, darunavir; IDV, indinavir; LPV, lopinavir; NFV, nelfinavir; NVP, nevirapine; SQV, saquinavir; 3TC, lamivudine. (**c**) H9 cells were transfected with control siRNA or ASK1-targeted siRNA for 48 h before infection of HIV-1. At 2 days after infection, cells were washed and additionally cultured for 24 h in the presence or absence of AZT (10 μM). Cells and supernatants were then harvested and analysed by western blotting against the indicated antibodies. We used AZT-resistant virus to avoid the effects of AZT carry-over from the culture supernatant of producer cells during the reverse transcription step in target cells. (**d**) TZM-bl reporter cells were infected with harvested and normalized virus to measure viral infectivity (*n*=3, mean±s.d.). Full images for all western blots analysis are shown in Supplementary Fig. 6.

Generally, MAP3Ks act as stress-responsive kinases that quickly activate downstream cascades by sensing various stimuli such as cytokines, hormones and anticancer drugs^36^. We wished to evaluate the pathophysiological significance of ASK1-mediated antiviral activity. Initially, we assessed whether approved antiretroviral drugs could induce ASK1 activity. Interestingly, the reverse transcriptase inhibitors azidothymidine/zidovudine (AZT) were found to induce ASK1 expression in peripheral blood mononuclear cells (PBMCs) and in the H9 CD4^+^ T-cell line (Fig. 5a,b). A previous report has indicated that the maximum serum AZT concentration after oral administration is ∼10 μM (ref. 37). We next assessed the possibility that AZT treatment at physiological concentrations would activate the ASK1–A3G axis in HIV-infected cells. H9 cells were transfected with either ASK1-targeted short interfering RNA (siRNA) or control siRNA and then infected with HIV. At 2 days after infection, the cells were washed and additionally cultured for 24 h at the presence or absence of AZT at 10 μM (Fig. 5c). In this experiment, we used an AZT-resistant virus harbouring reverse transcriptase mutations (T69G, K70R, L74I, K103N, T215F and K219Q)^38^,^39^ to minimize the carry-over effect of AZT from the cell culture supernatants of producer cells. We found that A3G incorporation into virions was enhanced by transient AZT treatment of control cells, but this was not the case in ASK1-depleted cells (Fig. 5c). In accordance with the results for A3G amounts in virions, the infectivity of viruses derived from AZT-treated cells was significantly reduced; this reduction was blocked by ASK1 depletion in virus-producer cells (Fig. 5d). Taken together, our data show that AZT can promote the antiviral activity of A3G by inducing ASK1.

## Discussion

**Figure 6:**
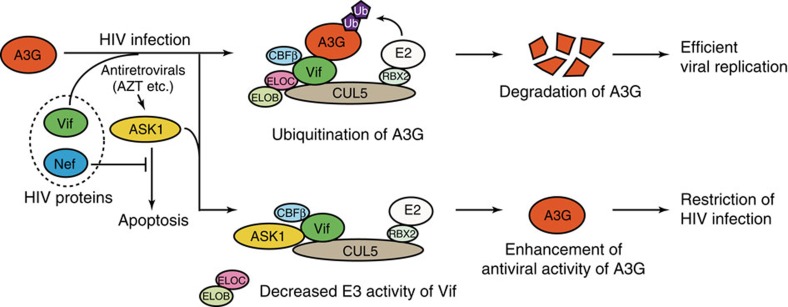
A proposed model for ASK1 enhancement of the antiviral activity of A3G. For efficient viral replication, Vif targets A3G for ubiquitination and proteasomal degradation by forming a stem cell factor-like E3 ubiquitin ligase complex composed of CUL5, ELOB/C and CBFβ. External agents such as AZT can induce ASK1 expression. ASK1 physically interacts with Vif and interferes with the formation of the Vif–E3 ubiquitin ligase complex. Consequently, the antiviral activity of A3G is restored and the virus replication is inhibited. Although another HIV accessory protein, Nef, inhibits the kinase activity of ASK1, this protein does not appear to alter the impact of ASK1 on Vif.

We here demonstrate that ASK1 is an AZT-inducible host factor that negatively regulates the Vif-mediated degradation of A3G to restore intrinsic antiviral immunity to HIV-1 (a proposed model is depicted in Fig. 6). Since our preliminary studies have suggested that several external stimuli can inhibit Vif-mediated A3G degradation, we here targeted human protein kinases as responders to the external stimuli that regulate the functionality of Vif. A fluorescence-based screen ultimately identified ASK1 as acting as an ‘anti-Vif factor' in terms of A3G protein stability. Indeed, cells overexpressing ASK1 showed a restored A3G antiviral function and rarely spread infectious viral particles in the secondary infection. Our study findings thus shed new light on the molecular link between ASK1 and Vif-mediated HIV-1 evasion of the host antiviral system and provide a better understanding of the role of a pre-existing antiretroviral drug in bolstering the host innate immune system.

Cellular regulatory mechanisms confer a sensitive, specific and robust response to external stimuli and initiate certain molecular events in cells. Such dynamic regulation is achieved through post-translational modifications (PTMs) including phosphorylation and ubiquitination. PTMs offer a dynamic way to regulate protein–protein interactions and protein activity, subcellular localization and stability^40^. Thus, virus–host protein interactions can also be modulated by PTMs as a response to external stimuli such as chemicals, growth factors and cytokines. In this regard, Mehle *et al*.^33^ reported that the phosphorylation of Vif blocks assembly of the Vif–E3 complex. Moreover, the NEDD8 ubiquitin-like protein modification pathway also regulates the function of Vif and/or Vpx–E3 complex^11^,^41^,^42^. Thus, many signalling pathways may antagonize the function of Vif or Vif–E3 complex to suppress Vif-mediated A3G degradation. Since the activity of protein kinases or ubiquitin ligases is basically regulated by the cellular context governed by external or internal signals, these results suggest that the functional interaction between viral accessary proteins and host restriction factors can be, at least in part, regulated by extracellular events.

ASK1 has been identified as MAP3K^43^ and is an effector of the external stimuli-triggered signalling that induces apoptosis^36^. Concordant with the role of ASK1 in virus infection, ASK1 is involved in the apoptosis of cells infected by influenza A virus^44^. Importantly, HIV-1 Nef is another ASK1-interacting protein that suppresses tumour necrosis factor-α-induced cell death in HIV-1-infected T-cell lines^34^. Although Kumar *et al*.^45^, using a luciferase assay system, recently reported a dynamic interaction between Nef and ASK1, this interaction was only marginally detectable in our hands by conventional protein–protein interaction analysis. Our current analyses demonstrate an alternative role for ASK1 in HIV-1 infection, with ASK1 interacting with Vif and negatively modulating the function of Vif in terms of E3 ligase activity. Importantly, the kinase activity of ASK1 was found in our analysis to be dispensable for Vif counteraction because (i) the kinase-dead mutant of ASK1 could still inhibit Vif-mediated A3G degradation, and (ii) Nef, a suppressor of ASK1 kinase activity, did not affect the function of ASK1 directed against Vif. However, a constitutively active ASK1 mutant (ΔN) seems to have a larger inhibitory effect on Vif than its wild-type counterpart in cell-based assays. This may be the result of a higher Vif-binding affinity of ΔN ASK1 compared with wild type. In fact, our data show that ASK1 directly bound to the BC-box motif of Vif and blocked the interaction between Vif and ELOB/C. Consequently, assembly of the components of the Vif–E3 ubiquitin ligase complex failed to degrade A3G. Our computational docking simulation of the Vif–ASK1 interaction predicted that the binding interface for ASK1 within Vif belongs to a conserved region that is considered to be essential for formation of the E3 complex. ASK1 may exploit this weak point of Vif to effectively suppress its action.

We further found an unexpected ability of antiretroviral AZT to restore antiviral immunity by suppressing Vif function via ASK1. AZT is a chemical variant of the natural nucleoside thymidine formed by the addition of an azido group, and is a widely used nucleoside inhibitor that arrests reverse transcript synthesis of viral DNA. AZT is transported to mitochondria^46^ and affects mitochondrial metabolism, causing mitochondrial dysfunction that generates oxidative stress in cells^47^. This internal stress may induce ASK1 to suppress the effects of Vif. Although the pathway(s) underlying ASK1 induction following AZT treatment are still unknown at present, *ASK1* gene analysis in future studies may elucidate the molecular mechanisms by which ASK1 senses drugs.

In our current study, we mainly analysed the function of overexpressed ASK1 in 293 cells and T-cell lines. Since ASK1 is an apoptosis-regulating protein^36^,^43^, it is not expressed or is expressed at very low levels in certain tissues and cells including PBMC and T cells at steady state. Our current data demonstrate that physiological concentrations of AZT stimulated the expression of ASK1 in T cells in which the ability of Vif to neutralize A3G was blocked (Fig. 5). Interestingly, this may not reflect a general effect of ASK1 to repress ELOB/C-binding proteins, since the activity of VHL was not impaired by ASK1 (Supplementary Fig. 2). While our study shows a distinct polypharmacological effect of AZT, there are still many unanswered questions about the physiological role and/or clinical relevance of the ASK1–Vif interaction. To address these important questions, longitudinal research and fine-grained assessment should be conducted in the future.

In conclusion, we here demonstrate that ASK1 is a novel Vif-binding protein that negatively regulates Vif-mediated A3G degradation. The interaction between HIV-1 accessory proteins and host restriction factors is a potential target for the development of new antiviral drugs. Understanding the molecular mechanism of this interplay will provide new insights into the preservation of the intrinsic antiviral system and may be useful for the future therapeutic treatment of HIV infection.

## Methods

### Plasmids

Human MAP3Ks, ELOB/C, CUL5 and CBFβ genes were amplified from the Mammalian Gene Collection complementary DNA (cDNA) library and subcloned into pcDNA-based vectors (Life Technologies, Gaithersburg, MD). The accession codes for the genes used are listed in Supplementary Table 1. Haemagglutinin (HA)-tagged human ASK1 expression vector and its deletion mutants (ΔN, ΔC, NT, KD, CT and K709M) have been described previously^48^,^49^,^50^,^51^. The ASK1 cDNAs were inserted into the pcDNA4/HisMax vector (Life Technologies) to obtain Xpress (XP)-tagged ASK1 or into the pRetroX-TRE3G vector (Clontech, Palo Alto, CA) to generate retroviral vector. Plasmids encoding HIV-1_NL4-3_ Vif^52^,^53^, human A3G^54^ and ubiquitin^55^ have been described. The Vif mutants were generated with PCR-based molecular cloning procedures. HIV-1_NL4-3_ Nef was amplified from a pNL4-3 molecular clone^56^ and subcloned into p3xFLAG-CMV-14 vector (Sigma-Aldrich, St Louis, MO). For *in vitro* protein synthesis, each cDNA was subcloned into pEU vector (CellFree Sciences, Ehime, Japan).

### Cells and viruses

HEK293, HEK293T (ATCC) and TZM-bl (NIH AIDS Reagent Program) cells were maintained in DMEM supplemented with 10% fetal bovine serum. CEM, CEMSS, H9, M8166 (NIH AIDS Reagent Program) and human PBMCs (purchased from Kurabo, Osaka, Japan) were cultured in RPMI containing 10% fetal bovine serum. To generate CEM-TetON-ASK1 or CEMSS-TetON-ASK1 cells, parental cells were co-infected with VSV-G-pseudotyped retroviral vectors expressing pRetroX-TRE3G-ASK1 and pRetroX-Tet3G (Clontech) and selected with G418 and puromycin. HIV-1 stocks were produced by transient transfection of HEK293T cells with the pNL4-3 (ref. 56) or AZT-resistant pNLGRINFQ molecular clones (NIH AIDS Reagent Program). Culture supernatants containing virus were collected at 48 h after transfection, filtered through a 0.45-μm Millex-HV filter (Merck Millipore, Billerica, MA) and immediately stored until use.

### Ubiquitination and immunoprecipitation analysis

HEK293 cells in six-well plates were transfected with vectors encoding XP-ASK1 (500 ng), HA-A3G (50 ng), Vif (100 ng) and Myc-ubiquitin (500 ng). Cells were treated with 2 μM MG132 (a proteasome inhibitor, Sigma-Aldrich) for 18 h before being harvested. At 48 h after transfection, cells were lysed with HBST buffer (10 mM HEPES (pH 7.4), 150 mM NaCl, 0.5% Triton-X-100) containing protease inhibitor Complete mini (Roche Diagnostics, Basel, Switzerland). Cell lysates were immunoprecipitated with EZview Red anti-HA Affinity Gel (Sigma-Aldrich) or 2 μg of anti-Vif antibody (Clone #319; NIH AIDS Reagent Program)^57^,^58^,^59^ mixed with protein G sepharose (GE Healthcare, Healthcare, Little Chalfont, UK) and bound proteins were analysed by western blotting as follows. Samples were loaded onto 10% or 3–15% gradient gels and blotted onto PVDF membranes (Merck Millipore). Membranes were probed with primary antibodies and horseradish peroxidase-conjugated secondary antibodies (GE Healthcare). The antibodies used including dilutions are listed in Supplementary Table 2. The proteins detected were visualized on a FluorChem digital imaging system (Alpha Innotech, San Leanardo, CA) and the band intensities were quantified with NIH ImageJ software.

### AlphaScreen and pull-down assays

The wheat germ cell-free protein production with pEU vectors and AlphaScreen analysis has been described previously^60^,^61^,^62^. In brief, DNA templates containing a biotin-ligating sequence or FLAG epitope were amplified by split-PCR with pEU-based vectors and corresponding primers and then used with the GenDecoder protein production system (CellFree Sciences). In this study, Vif proteins were co-expressed with untagged CBFβ to stabilize the conformation of Vif protein^28^,^63^. For the *in vitro* competitive pull-down assays, recombinant biotinylated Vif was pre-mixed with untagged ASK1 at a molar ratio of 1:1, 1:2, 1:4 or 1:8 for 5 min at room temperature and then mixed with equivalent amounts of FLAG-tagged CUL5, ELOB and ELOC proteins. After 1 h at 26 °C, the mixture was processed for pull-down with streptavidin-coated magnetic beads (Merck Millipore). Bound proteins were detected with western blot analysis.

### Transfection-based single-round infection assays

HEK293 cells in six-well plates were cotransfected with pNL4-3ΔEnv-GFP or pNL4-3ΔEnvΔVif-GFP (1 μg) and with vectors encoding VSV-G (400 ng), ASK1 (250 ng) and A3G (25 ng) and cultured for 2 days. The culture supernatants and cell lysates were subjected to western blotting analysis. The p24 antigens in supernatants were measured with an ELISA kit (Zepto Metrix, Buffalo, NY), and M8166 cells were infected with normalized viruses (1 or 5 ng of p24 antigen) in 24-well plates for 24 h. Infectivity was calculated by counting the numbers of GFP-positive cells.

### Multicycle replication assays

CEM, CEMSS or their derivative cells (10^5^ cells) were infected with HIV-1_NL4-3_ (50 ng of p24 antigen) with or without 1 μg ml^−1^ Dox. Viral supernatants were collected periodically and p24 levels were measured as described above. In AZT experiments, H9 cells (10^6^ cells) were transfected with 200 pmol ASK1-targeted siRNA (HSS106458; Life Technologies) or control siRNA using Neon nucleofection system (Life Technologies) at 48 h before infection. Cells were then infected with 50 ng of AZT-resistant HIV-1_NLGRINFQ_. After 2 days, cells were washed four times with PBS and then treated with 10 μM AZT (Sigma-Aldrich) for 24 h. The culture supernatants and cell lysates were then harvested and subjected to western blotting or infectivity analysis. Infectivity was calculated by measuring LTR-driven luciferase activity of TZM-bl indicator cells infected with normalized viruses.

### G-to-A hypermutation assays

Cellular DNA from HIV-infected cells was extracted with a QIAamp DNA Mini Kit (Qiagen, Venlo, Netherlands) according to the manufacturer's instructions. The extracted DNA was amplified using a primer pair HIV-1F (5′- AGGCAGCTGTAGATCTTAGCCACTT -3′) and HIV-1R (5′- GGTCTGAGGGATCTCTAGTTAC -3′) and cloned into pGEM-T vector (Promega, Madison, WI). Eight clones containing a 650-bp DNA fragment (a portion of nef and 3′ long terminal repeat region) were sequenced and the numbers of mutations in the viral genomes were analysed.

### Protein structure prediction and docking simulation

The structure models for the CT domain of human ASK1 (ASK1 CT; 955–1,374 amino acids) were obtained by I-TASSER software v2.1 (refs 64, 65) or Molecular Operating Environment software using templates (such as PDB accession codes 1OQY, 1IQC and 1QBK) for the structural assembly simulations. To obtain the docking structure of Vif (PDB accession code 4N9F) and ASK1 CT, we used the docking simulation server ClusPro 2.0 (refs 66, 67).

### Statistical analysis

All graphs present the mean and s.d. The statistical significance of differences between two groups was tested by two-tailed unpaired *t*-test with Prism 6 software (GraphPad, La Jolla, CA). A *P* value of <0.05 was considered statistically significant.

## Author contributions

All authors contributed extensively to the work presented in this paper. K.M. designed the study, performed the experiments, analysed the data and wrote the manuscript. S.M. and R.M. performed the experiments and analysed the data. K.K., M.Y. and H.S. performed the modelling and docking simulation analysis. A.M., K.S., H.I. and A.T.-K. provided materials and discussed the data. A.K., H.K., T.T. and N.Y. analysed and discussed the data. A.R. designed and supervised the study, analysed the data and wrote the manuscript.

## Additional information

**How to cite this article:** Miyakawa, K. *et al*. ASK1 restores the antiviral activity of APOBEC3G by disrupting HIV-1 Vif-mediated counteraction. *Nat. Commun.* 6:6945 doi: 10.1038/ncomms7945 (2015).

## Supplementary Material

Supplementary Information Supplementary Figures 1-7, Supplementary Tables 1-2 and Supplementary References
