# Supplementary material for: ASK1 restores the antiviral activity of APOBEC3G by disrupting HIV-1 Vif-mediated counteraction
Source: Nat Commun. 2015 Apr 22;6:6945. doi: 10.1038/ncomms7945 (PMC4423214; doi:10.1038/ncomms7945)
Supplement: Supplementary Information — Supplementary Figures 1-7, Supplementary Tables 1-2 and Supplementary References [file ncomms7945-s1.pdf]

Supplementary Figure 1

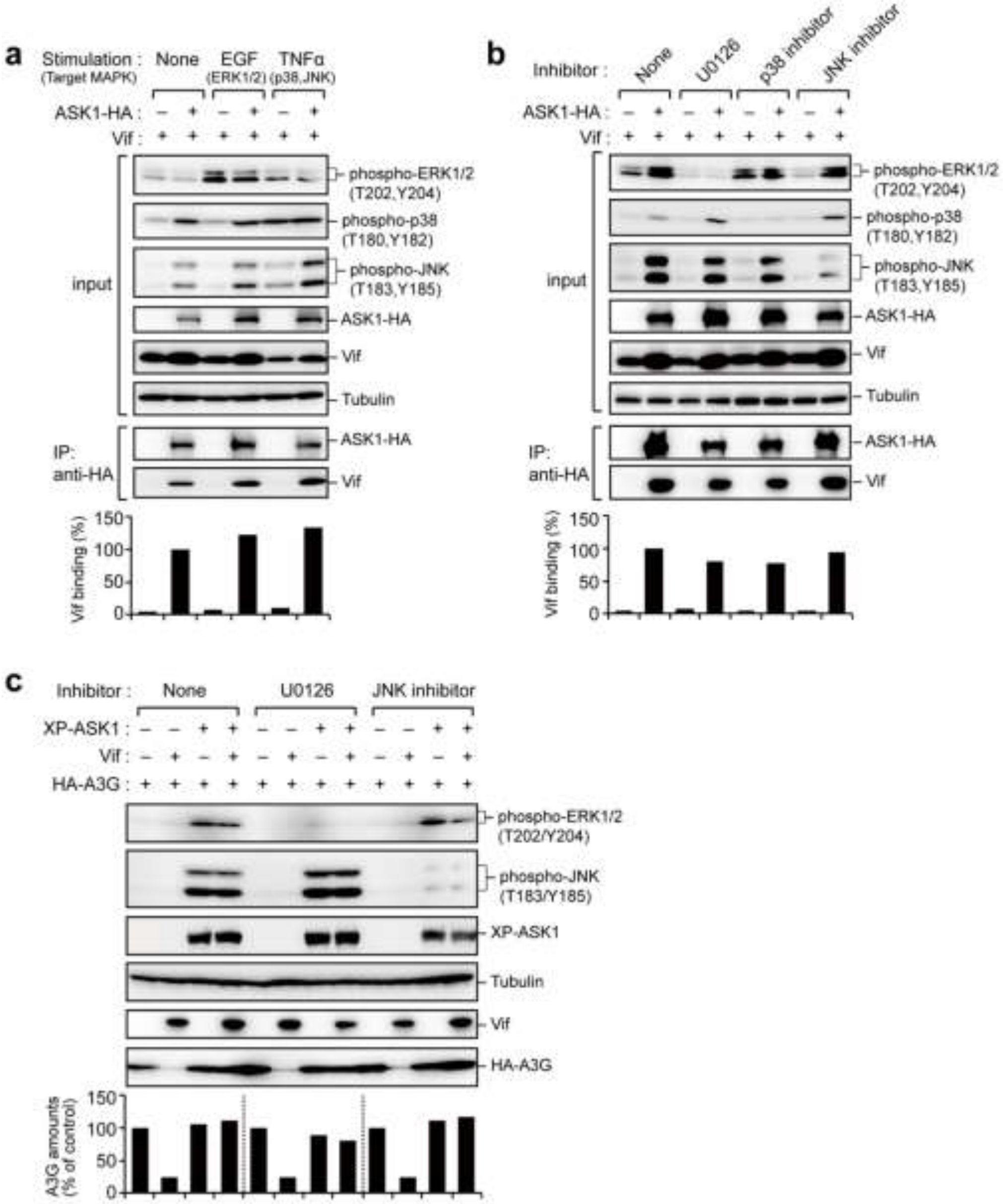

Supplementary Figure 1. Neither the activation nor suppression of the MAPK pathway affects the ASK1/Vif interaction.

(a, b) HEK293 cells were cotransfected with plasmids encoding ASK1-HA and Vif. Cells were stimulated with EGF (10 µM) or TNFα (10 ng ml^{-1}) for 30 min (a) or treated with U0126 (10 µM), p38 or JNK inhibitor (20 µM) for 16 h (b) before being harvested. Cell lysates were then immunoprecipitated with anti-HA antibody and the bound proteins were analyzed by western blotting. Note that ASK1 expression activates p38 and JNK MAPKs, as reported previously[1]. The bar charts below the blot indicate the precipitated Vif levels determined by densitometry. (c) MAPK inhibitors do not influence Vif inactivation by ASK1. HEK293 cells were cotransfected with plasmids encoding XP-ASK1 (500 ng), HA-A3G (10 ng) and Vif (100 ng). Cells were treated with U0126 (10 µM) or JNK inhibitor (20 µM) for 16 h before being harvested. Protein expression was detected by western blot. The bar chart below the blot indicates the HA-A3G levels determined by densitometry. Full images for all western blots analysis are shown in Supplementary Fig. 7.

Supplementary Figure 2

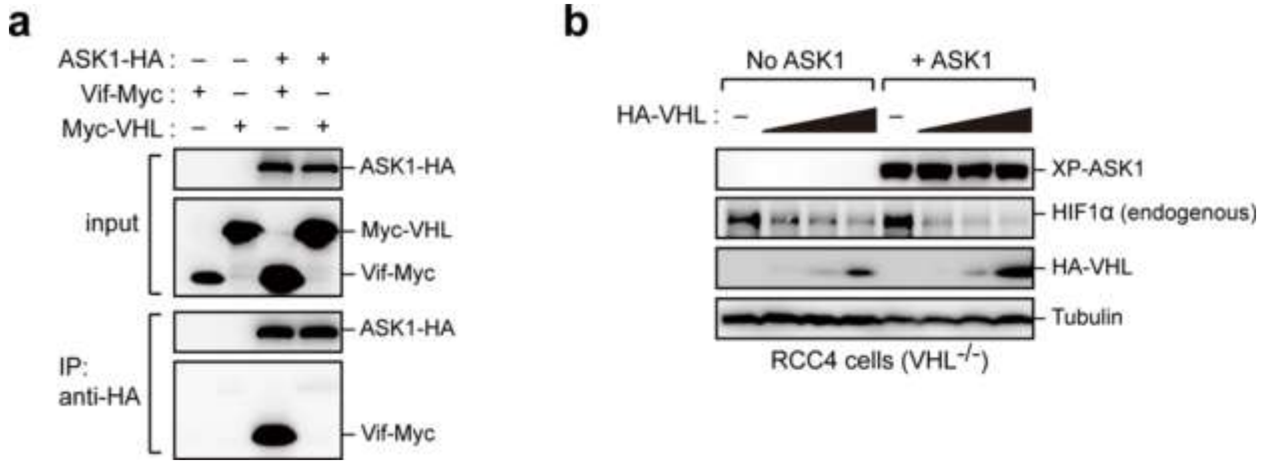

Supplementary Figure 2. ASK1 does not influence VHL-induced HIF1α degradation.

(a) ASK1 interacts with Vif, but not VHL. HEK293 cells were cotransfected with plasmids encoding ASK1-HA and either Vif-Myc or Myc-VHL. Cell lysates were immunoprecipitated with anti-HA antibody and bound proteins were detected by western blot. (b) RCC4 (VHL-negative) cells were cotransfected with plasmids encoding XP-ASK1 (500 ng) and HA-VHL (0, 100, 200 or 500 ng). Protein expression was detected by western blot. Full images for all western blots analysis are shown in Supplementary Fig. 7.

Supplementary Figure 3

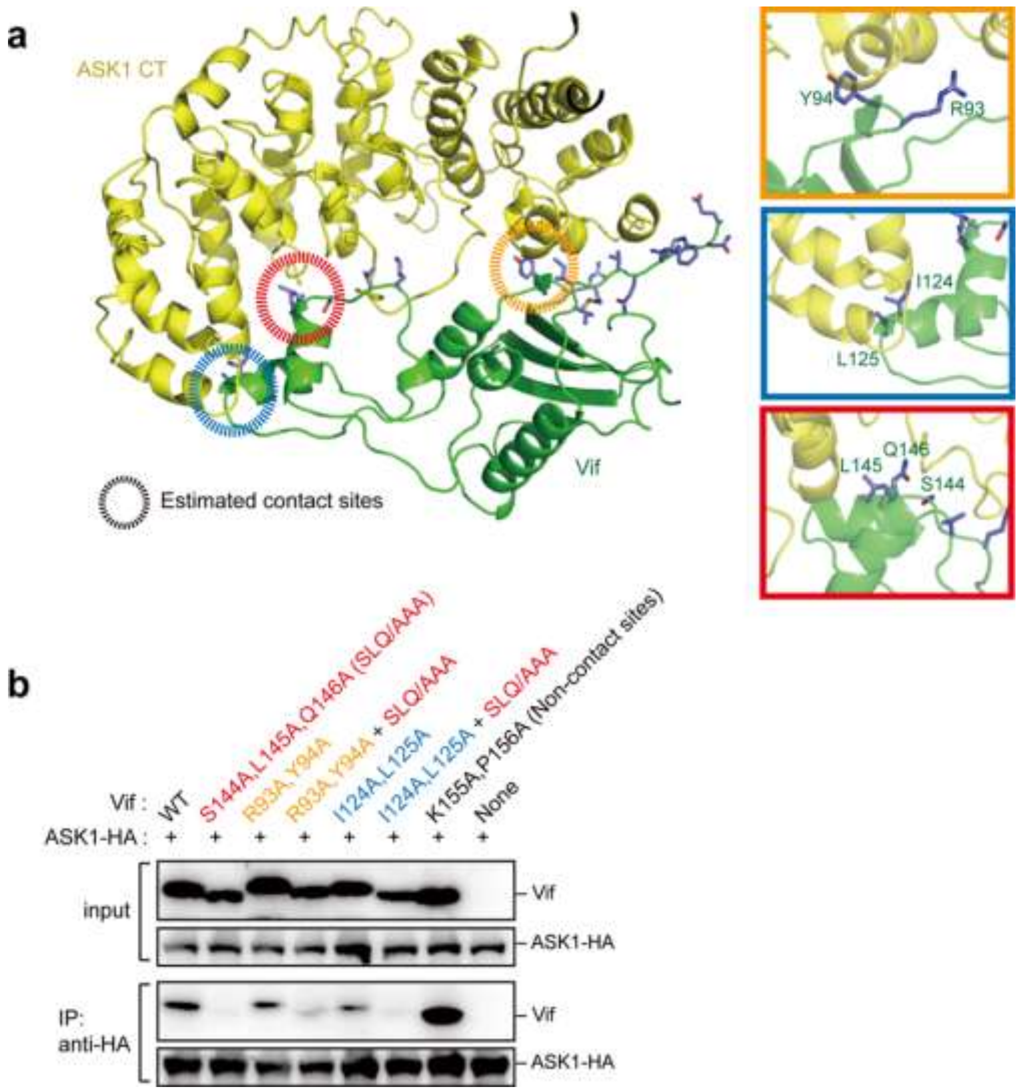

Supplementary Figure 3. The BC-Box motif of Vif is critical for ASK1 binding.

(a) Model of the ASK1 CT in complex with Vif. Three estimated contact sites are shown. (b) HEK293 cells were cotransfected with plasmids encoding ASK1-HA and Vif or the indicated Vif mutants. Cell lysates were immunoprecipitated with anti-HA antibody and bound Vif proteins were detected by western blot. Full images for all western blots analysis are shown in Supplementary Fig. 7.

Supplementary Figure 4

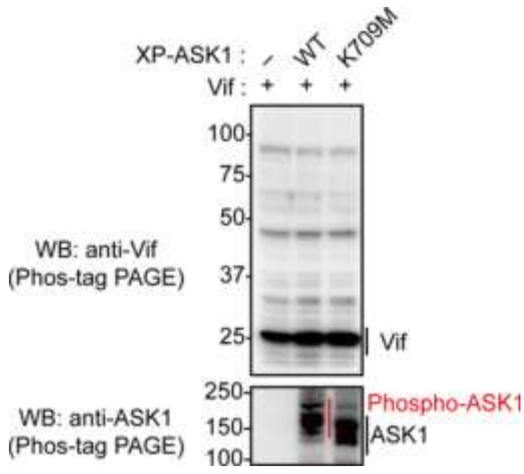

Supplementary Figure 4. ASK1 seems not to induce Vif phosphorylation.

HEK293 cells were cotransfected with plasmids encoding Vif and either WT ASK1 or its kinase-dead mutant (K709M). Cell lysates were then subjected to Phos-tag PAGE analysis. This method enables the visualization of the phosphorylation status of a protein as a distinct band shift[2, 3]. Blots were incubated with anti-ASK1 and anti-Vif antibodies. Full images for all western blots analysis are shown in Supplementary Fig. 7.

Supplementary Figure 5

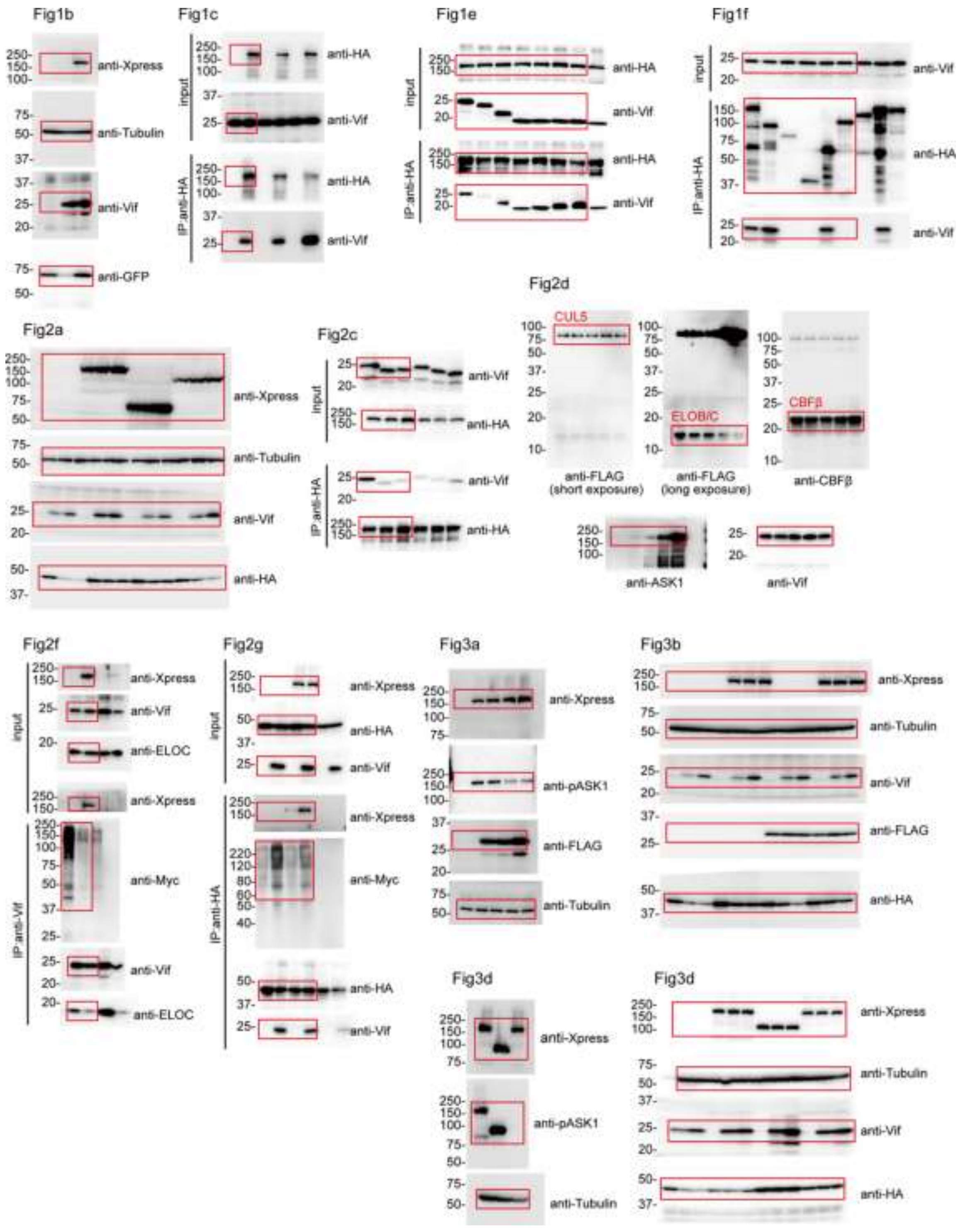

Supplementary Figure 5. Full images of the western blot analysis presented in Figure 1, 2, and 3.

Supplementary Figure 6

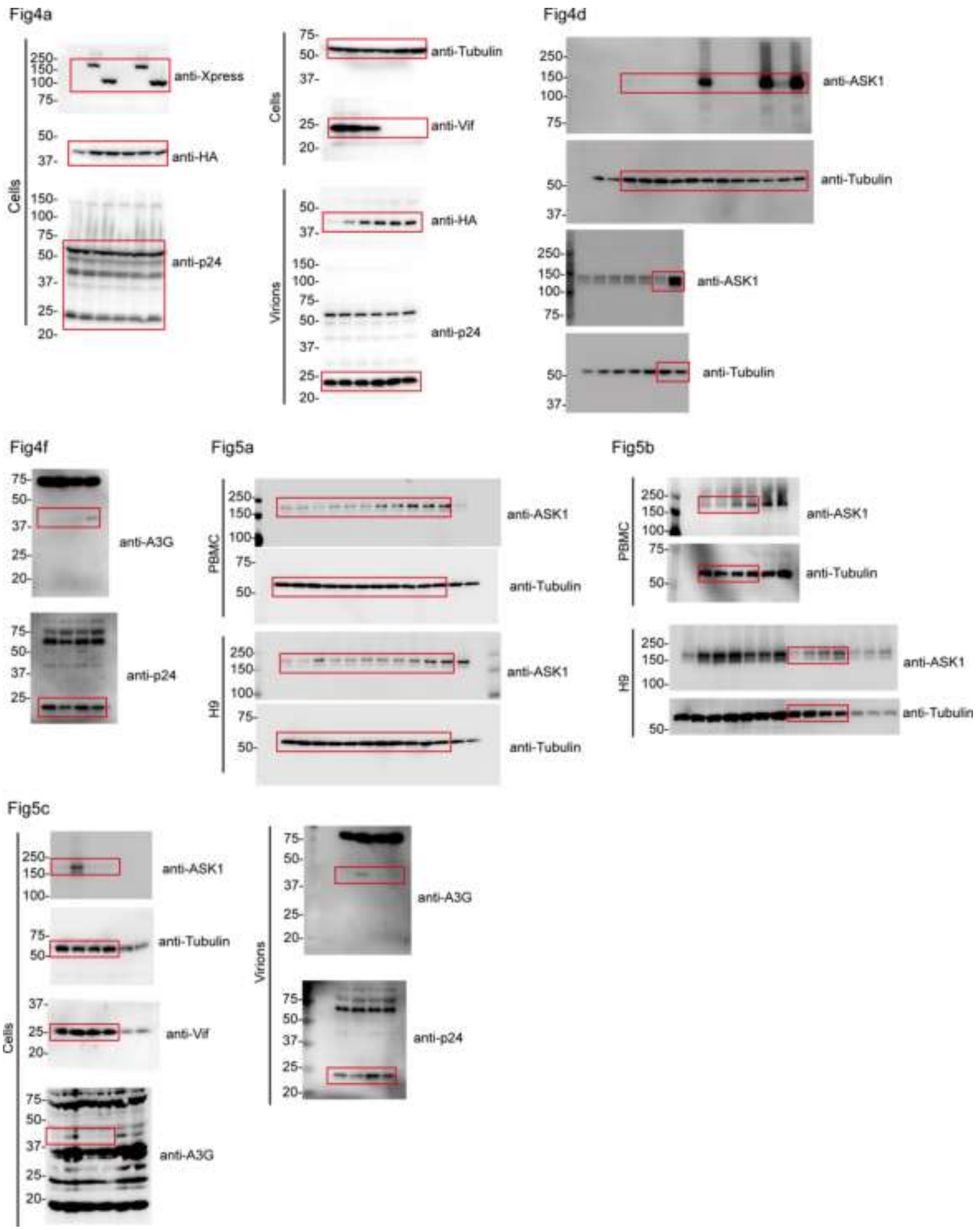

Supplementary Figure 6. Full images of the western blot analysis presented in Figure 4 and 5.

Supplementary Figure 7

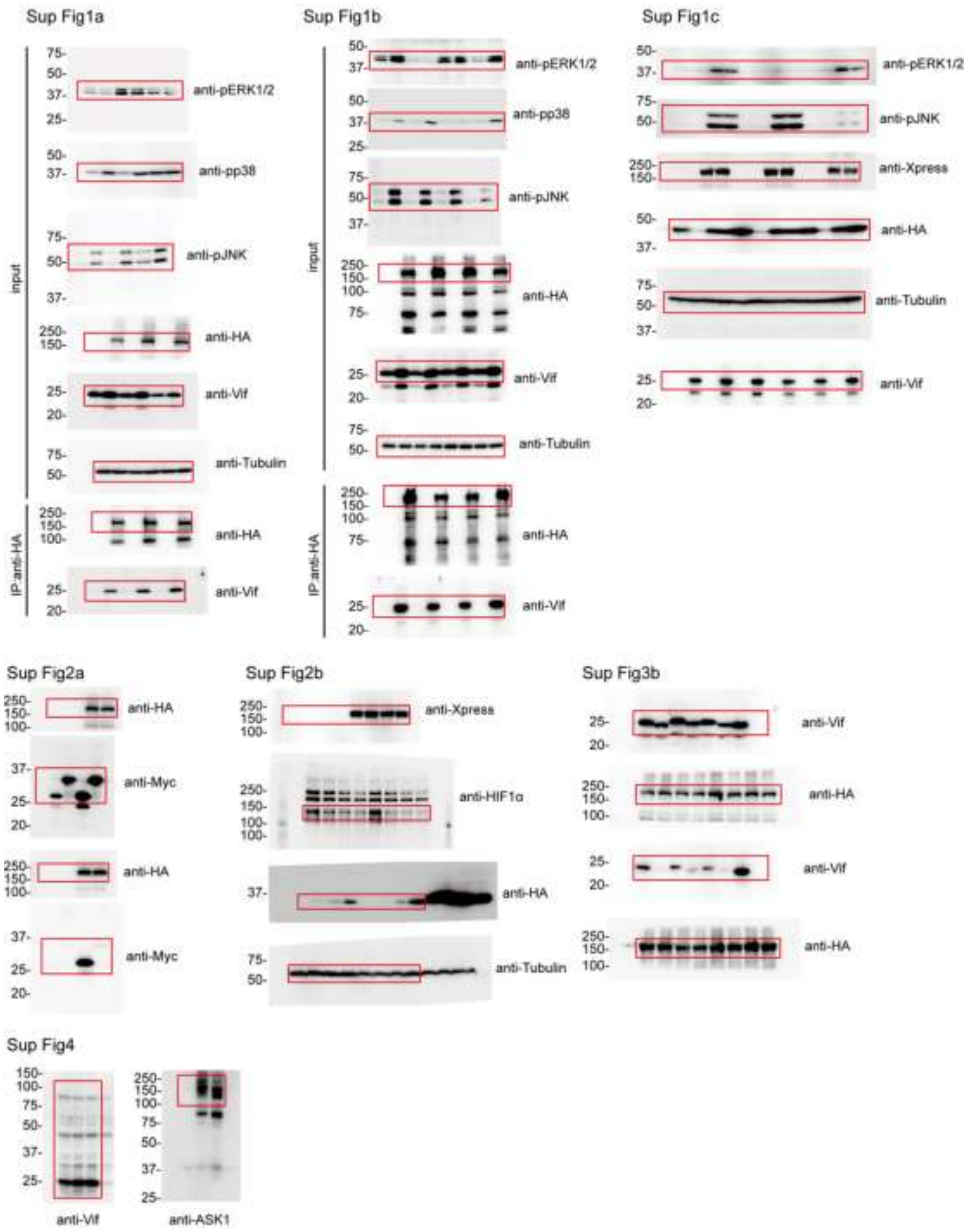

Supplementary Figure 7. Full images of the western blot analysis presented in Supplementary Figures.

Supplementary Table 1

Gene	Accession	Primer pair (5' - 3')
**MEKK2**	BC136293	AGATTACGCTGCTAGCATGGATGATCAGC
		TAGCCTCCCCCGTTTAAACCTAGTGATAAT
**MEKK4**	BC143735	AGATTACGCTGCTAGCATGAGAGACGCCAT
		TAGCCTCCCCCGTTTAAACTCACCCACAAA
**TAK1**	BC017715	AGATTACGCTGCTAGCATGTCTACAGCCTCTGCCG
		TAGCCTCCCCCGTTTAAACTCATGAAGTGCCTTGTCG
**TPL2**	BC104833	AGATTACGCTGCTAGCATGGAGTACATGAG
		TAGCCTCCCCCGTTTAAACTCAGCCATATT
**MLK3**	BC064543	AGATTACGCTGCTAGCATGGAGCCCTTGAA
		TAGCCTCCCCCGTTTAAACTCAAGGCCCCG
**ZPK**	BC050050	AGATTACGCTGCTAGCATGGCTTGCCTCCA
		TAGCCTCCCCCGTTTAAACTCAGGGGATCA
**NIK**	BC035576	AGATTACGCTGCTAGCATGGCAGTGATGG
		TAGCCTCCCCCGTTTAAACTTAGGGCCTG
**TAOK2**	BC144344	AGATTACGCTGCTAGCATGCCAGCTGGGGG
		TAGCCTCCCCCGTTTAAACCTACCTCCA
**ELOB**	BC013306	AGATTACGCTGCTAGCATGGACGTGTTCCTC
		TAGCCTCCCCCGTTTAAACTCACTGCACGGCTTG
**ELOC**	BC013809	AGATTACGCTGCTAGCATGGATGGAGAGGAG
		TAGCCTCCCCCGTTTAAACTTAACAATCTAAGAAG
**CUL5**	BC063306	AGATTACGCTGCTAGCATGGCGACGTCTAATC
		TAGCCTCCCCCGTTTAAACTTATGCCATATATATG
**CBFβ**	BC018509	AGATTACGCTGCTAGCATGCCGCGCGTCGTG
		TAGCCTCCCCCGTTTAAACCTAGGGTCTTGTTGTC

Supplementary Table 1. Accession numbers and specific primers of genes used in this study.

Supplementary Table 2

Antibody	Source (Catalog number)	Dilution
**Xpress**	Life Technologies (R910-25)	1:10000
**α-Tubulin**	Sigma-Aldrich (T6199)	1:5000
**HIV-1 Vif**	NIH AIDS Reagent Program (6459)	1:2000
**GFP**	Roche (11814460001)	1:1000
**HA**	Roche (11867423001)	1:1000
**FLAG**	Sigma-Aldrich (F3165)	1:10000
**CBFβ**	GeneTex (GTX106820)	1:1000
**ASK1**	Abcam (ab45178)	1:1000
**ELOC**	Santa Cruz Biotechnology (sc-1559)	1:500
**Myc**	Cell Signaling Technology (2272)	1:1000
**Phospho-ASK1**	Commercially unavailable[4,5]	1:2000
**HIV-1 Gag p24**	NIH AIDS Reagent Program (3537)	1:1000
**APOBEC3G**	NIH AIDS Reagent Program (9968)	1:1000
**Phospho-ERK1/2**	Cell Signaling Technology (4370)	1:1000
**Phospho-p38**	Cell Signaling Technology (4631)	1:1000
**Phospho-pJNK**	Cell Signaling Technology (9251)	1:1000
**HIF1α**	BD Biosciences (610958)	1:1000
**HRP-conjugated anti-mouse IgG**	GE Healthcare (NA931)	1:10000
**HRP-conjugated anti-rabbit IgG**	GE Healthcare (NA934)	1:10000

Supplementary Table 2. Antibodies used in this study.

Supplementary References

1. Ichijo H, *et al.* Induction of apoptosis by ASK1, a mammalian MAPKKK that activates SAPK/JNK and p38 signaling pathways. *Science* **275**, 90-94 (1997).
2. Kinoshita, E. Kinoshita-Kikuta, K. Takiyama, T. Koike. Phosphate-binding tag, a new tool to visualize phosphorylated proteins. *Molecular & Cellular Proteomics* **5**, 749–757 (2006).
3. Kinoshita, E. Kinoshita-Kikuta, T. Koike. Separation and detection of large phos-phoproteins using Phos-tag SDS-PAGE. *Nature Protocol* **4**, 1513–1521 (2009).
4. Saitoh M, et al. Mammalian thioredoxin is a direct inhibitor of apoptosis signal-regulating kinase (ASK) 1. *The EMBO journal* **17**, 2596-2606 (1998).
5. Tobiume K, Saitoh M, Ichijo H. Activation of apoptosis signal-regulating kinase 1 by the stress-induced activating phosphorylation of pre-formed oligomer. *Journal of Cellular Physiology* **191**, 1091–1099 (2002).
